# The Value of Education to the Dentist

**Published:** 1907-06

**Authors:** J. M. Quattlebaum


### THE VALUE OF EDUCATION TO THE DENTIST.

J. M. QUATTLEBAUM, D. D. S.

S. C. State Dental Association, 1906.

One of the basic characteristics of human nature is its social instinct, which has ever held a determining influence on human life. This is to men what the gregarious instinct is to certain higher order of animals. Man seeks his fellow man, and his greatest delight is human fellowship. The garden of Eden with its delights was insufficient to supply the wants of its lone inhabitant. The completion of its happiness demanded social fellowship. Then was woman created and given to man as a companion. The family thus formed became the unit of society and by the multiplication of this unit have been created all the institutions, social, civil and religious that mark the progress of the race from barren and unsatisfying individualism to that unity and community which is the source of our greatest happiness and strength. This principle grew with the growth of civilization, so that we find the tract of human progress is marked by institutions that are meant to draw men into closer relations for their mutual good.

The Dental Association of South Carolina is only an integral of that complex organization which has grown up in the gradual evolution of civilization and enlightenment. Once each year it is our pleasure to retire to some place apart, where we may compare experiences, confer as to our common interest and inspire one another to strive for higher ideals. To our Association is committed in a large measure the interests of an honarable and useful profession, and its meetings should not be treated with indifference. Here as in the family and state the good result attained depends upon the faithfulness of each member.

This then must stand as my apology, if apology is needed for offering my small contribution on the subject assigned me, viz.: Education.

This is an old theme. We can hope to say nothing that is really new but may do nothing more than "thrash over the old straw," but success is gained only by repeated efforts. We need to be reminded of what we knew before, it is necessary then in this presence to re-emphasize the value of education. Lord Bacon sums up this in the nutshell "knowledge is power." Education is light and it is capital in every vocation of life. Education is the only power to think, and this is the mightiest power in the universe, because it guides and controls all other powers. Benj. Franklin's brain harnessed the lightning and transformed it into an obedient servant. Robt. Stevenson's brain brought steam under tribute and made the steam engine man's steed and beast of burden. However, there is no need of multiplying examples, no one doubts that brain is mightier than brawn. There may have been a time when the farmer was thought of only as a man of muscle, able to guide the plow or wield the axe, now the real farmer is one who mixes brain with his soil and who brings the product of human invention and the forces of nature to do the work once done by brute force.

Is he a dentist who can extract a tooth and make a plate? Is there nothing more for him to know? Is the technical skill the acme of his profession? Certainly not. Such a conception as this brings the profession down to the level of the mechanical arts and makes the Dentist only an artisan.

What we are attempting to say does not relate primarily to Dental Education as such. Of course, there must be some acquirements in this knowledge, but we refer rather to that cultivation and development of the mental nature, which we maintain is an essential preparation for the acquirement of knowledge and skill. Our plea then is a broader culture as a foundation for professional training. A good measure of such intellectual culture is demanded if Dentistry is to occupy the place among the professions which it is entitled to. Without it Dentistry becomes only a craft limited to narrow bounds by the want of mental capacity by those who practice it. The field is limitless in its possibilities of advancement to the practioner who knows how to think. The lack of this power leaves him an imitator of those who have thought before him. There is much foolish sentiment indulged in by those who disparage theory and magnify the practical. The world's greatest discoverers and inventors have been theorists. They have gone ahead of the plodding masses and blazed new paths for them.

All progress then in our noble profession depends upon a broader and deeper intellectual training, not simply in theoretical and practical Dentistry, but in all branches of knowledge that strengthen the mind. The power to think is worth a thousand thoughts. You cannot make a silk purse out of a sow's ear. No more can you make an accomplished Dentist out of an intellectual vacuum, though you try to fill it up by three years of instruction in the science and practice of Dentistry.

